# SDNN-PPI: self-attention with deep neural network effect on protein-protein interaction prediction

**DOI:** 10.1186/s12864-022-08687-2

**Published:** 2022-06-27

**Authors:** Xue Li, Peifu Han, Gan Wang, Wenqi Chen, Shuang Wang, Tao Song

**Affiliations:** College of Computer Science and technology, China University of Petroleum (East China), Qingdao, China

**Keywords:** Protein-protein interactions, Deep learning, Deep neural network, Self-attention

## Abstract

**Background:**

Protein-protein interactions (PPIs) dominate intracellular molecules to perform a series of tasks such as transcriptional regulation, information transduction, and drug signalling. The traditional wet experiment method to obtain PPIs information is costly and time-consuming.

**Result:**

In this paper, SDNN-PPI, a PPI prediction method based on self-attention and deep learning is proposed. The method adopts amino acid composition (AAC), conjoint triad (CT), and auto covariance (AC) to extract global and local features of protein sequences, and leverages self-attention to enhance DNN feature extraction to more effectively accomplish the prediction of PPIs. In order to verify the generalization ability of SDNN-PPI, a 5-fold cross-validation on the intraspecific interactions dataset of Saccharomyces cerevisiae (core subset) and human is used to measure our model in which the accuracy reaches 95.48% and 98.94% respectively. The accuracy of 93.15% and 88.33% are obtained in the interspecific interactions dataset of human-Bacillus Anthracis and Human-Yersinia pestis, respectively. In the independent data set Caenorhabditis elegans, Escherichia coli, Homo sapiens, and Mus musculus, all prediction accuracy is 100%, which is higher than the previous PPIs prediction methods. To further evaluate the advantages and disadvantages of the model, the one-core and crossover network are conducted to predict PPIs, and the data show that the model correctly predicts the interaction pairs in the network.

**Conclusion:**

In this paper, AAC, CT and AC methods are used to encode the sequence, and SDNN-PPI method is proposed to predict PPIs based on self-attention deep learning neural network. Satisfactory results are obtained on interspecific and intraspecific data sets, and good performance is also achieved in cross-species prediction. It can also correctly predict the protein interaction of cell and tumor information contained in one-core network and crossover network.The SDNN-PPI proposed in this paper not only explores the mechanism of protein-protein interaction, but also provides new ideas for drug design and disease prevention.

## Introduction

Proteins are organic macromolecules made up of amino acids, which are essential components of cells and sustain life activities. They play an important role in biology by linking various important physiological activities of cells to PPIs [1], enabling a range of life activities such as apoptosis and immune response. In recent years, a large number of high-throughput experimental methods have emerged to study PPIs, such as yeast two-hybrid screening [2], mass spectrometry [3], hybridization methods [4], immunoprecipitation [5] and protein microarrays [6]. However, all of these are based on biological and chemical experiments, which require a lot of manpower, financial and time resources. Therefore, artificial intelligence-based computational methods have emerged in bioinformatics [7, 8] and become quite prevalent predicting the interaction of proteins with other biological macromolecules [9, 10]. Especially in PPIs, there are abundant amino acid sequence information data, which is sufficient to establish PPIs prediction calculation model [11]. A growing number of researchers have been attracted by the aforementioned methods. The basic steps of PPIs prediction based on protein sequence consist of two parts: protein coding method and machine learning model.

With the rapid development of machine learning techniques [12–14] and the refinement of neural networks [15–18], some machine learning-based and sequence-based models have been presented for PPIs prediction. Shen et al. [19] first employed conjoint triad (CT) to extract features from protein sequences and predicted PPIs through support vector machine model incorporating kernel function with 83.9% accuracy. Guo et al. [20] proposed auto covariance (AC) to extract information from protein sequences and used support vector machine model to predict PPIs in the Saccharomyces cerevisiae dataset with 88.09% accuracy. Yang et al. [21] proposed local descriptors (LD) to represent protein sequences and successfully predicted potential PPIs on Saccharomyces cerevisiae (core subset) dataset by implementing K-neighbor model. You et al. [22] utilized four categories of protein sequence information (AC, CT, LD, MAC) to encode proteins as feature vectors focusing on dimensionality reduction and proposed a new hierarchical PCA-EELM (principal component analysis-integrated extreme learning machine) model to predict protein interactions. In 2014, Barman et al. [23] used support vector machine, Naive Bayes and random forest based on 5-fold cross-validation to complete the host-pathogen interaction prediction. In 2016, An et al. [24] jointly proposed a new computational method called RVM-BiGP, combining the relevance vector machine (RVM) model and Bi-gram probabilities (BiGP), to efficiently handle imbalanced protein interaction datasets. In 2018, Goktepe et al. [25] adopted PCA to fuse PSSM, Bi-gram, AAC, pseudo-amino acid (PseAAC) and weighted jump-order joint triple to obtain approximate features, then used SVM to complete PPIs prediction. Song et al. [26] used position specific scoring matrix (PSSM) to obtain evolutionary information and proposed a new feature fusion algorithm, which could combine discrete cosine transform (DCT), fast Fourier transform (FFT) and singular value decomposition (SVD). In 2019, Chen et al. [27] extracted features from PseAAC, autocorrelation descriptor (AD), CT and LD by elastic network, and predicted PPI in several datasets with the help of LightGBM network. In 2020, Yu et al. [28] proposed a combination of PseAAC, pseudo-position-specific scoring Matrix (PsePSSM), reduced sequence and index-vectors (RSIV), and AD to encode protein sequences for potential PPIs on Saccharomyces cerevisiae (core subset) dataset through GTB-PPI model.

Although machine learning methods can make predictions based on best fitting models, it is still open to some limitations on effectively learning the eigenvalues at a deep level. In recent years, deep learning architectures [8, 29–32] provide strong support for solving relevant problems in bioinformatics. In 2017, Wang et al. [33] extracted protein sequence features from PSSM, and reconstructed them through stacked auto-encoder. After that, prediction was completed with the help of a new probabilistic classification vector machine (PCVM). Du et al. [34] proposed a deep neural network model, DeepPPI, to improve the performance of PPIs prediction using AAC, gradient tree boosting (DC), LD and other protein transformations where demonstrated the superiority of the model on several datasets. Wang et al. [35] combined Deep Neural Networks (DNNs) with a new local composition ternary description (LCTD) feature representation, and proposed DNN-LCTD method to predict the PPIs on Saccharomyces cerevisiae (core subset) dataset with the accuracy of 93.12%. In 2018, Hashemifar et al. [36] efficiently combined deep Siamese-like convolutional neural networks and random projection to construct DPPI model for predicting PPIs by associating with protein evolutionary information. In 2019, Zhang et al. [37] proposed a deep model called EnsDNN, which extracted protein interaction information from AC, LD and multi-scale continuous and discontinuous local descriptors (MCD) which achieved 95.29% accuracy in Saccharomyces cerevisiae (core subset) dataset. You et al. [38] proposed a highly efficient method to detect PPIs by integrating a new protein sequence substitution matrix feature representation and ensemble weighted sparse representation model classifier. Yao et al. [39] designed a new protein sequence representation method, Res2vec, and combined effective feature embedding with deep learning techniques to develop the DeepFE-PPI framework, which achieved good performance in PPIs prediction. In 2020, Li et al. [40] represented proteins using AC, CT, LD, PseAAC, and built Ensemble model to complete PPIs prediction work. In 2021, Yu et al. [41] used PseAAC, AD, multivariate mutual information (MMI), composition-transition-distribution (CTD), amino acid composition PSSM (AAC-PSSM), and dipeptide composition PSSM (DPC-PSSM) to construct the pattern of GcForest-PPI.

Inspired by the above discussion, this paper proposes a protein-protein interaction prediction method, SDNN-PPI. Firstly, protein sequence information is encoded with AAC, CT, and AC. Second of all, in order to carry out effective feature extraction, the deep neural network combined with self-attention method is conducted to adjust the weight of the sequence and further emphasize the key features, so as to establish a network model to fully extract protein sequence information. Eventually, 5-fold cross-validation approach is applied in 2 intraspecies, 2 interspecies, and 4 independent datasets. All of which achieved high accuracy rates. To further evaluate the merits of the model, the effectiveness of the method is tested on one-core network and crossover network. The experimental results show that SDNN-PPI outperforms other state-of-the-art methods and is highly competitive.

## Materials and methods

### Data sets

In this study, multiple high-confidence PPI datasets were used to measure the performance of SDNN-PPI, including the intraspecific datasets Saccharomyces cerevisiae core subset (S.cerevisiae core subset) [20] and Human [38], the interspecific dataset Human-Bacillus Anthracis (Human-B.Anthracis) [42] and the Human-Yersinia pestis (Human-Y.pestis) [42]. The composition of the four datasets is shown in Table 1. In addition, four independent datasets [27] including Caenorhabditis elegans (C.elegans), Escherichia coli (E.coli), Homo sapiens (H.sapiens) and Mus musculus (M.musculus) are tested for PPIs. And the predictive performance of the method is further validated on two significant PPI networks [41]. One is the one-core CD9 network, which contains 16 PPIs, and the other is crossover network, which consists of 96 PPIs. There is also a data set, Saccharomyces Cerevisiae[34], for independent test experiments. In addition, to ensure the balance of positive and negative samples in the dataset, the same number of randomly selected negative samples is in the same amount as positive samples meaning the ratio of positive to negative samples was 1:1.

**Table 1:** Compositions of the four benchmark data sets

Data sets	Interaction pairs	Noninteraction pairs	Protein pairs
S.cerevisiae(core subset)	5594	5594	11188
Human	3899	4262	8161
Human-B.Anthracis	3094	9500	12594
Human-Y.pestis	4097	12500	16597
Saccharomyces cerevisiae	17257	48594	65551

### Feature extraction techniques

Since the length of the protein sequence is different, the input to the neural network used in the experiment is fixed. The protein sequences of different lengths have to be transformed into feature vectors of fixed length when they are input into network layers. In this paper, the feature fusion strategy is used to convert protein sequences into feature vectors based on AAC, CT and AC. AAC has the advantage of obtaining the proportion of each amino acid in the entire protein sequence from a global perspective. CT regards any continuous three amino acids as a unit, and puts the characteristics of amino acids and their adjacent amino acids into consideration, but ignores the information of amino acid discontinuous fragments. In terms of physicochemical properties, AC extracts not only discontinuous fragment information, but also the interaction features of long-distance amino acids by considering the adjacent effects of amino acids. In summary, this method extracts amino acid global features through ACC, and then uses CT to reduce the defect of few short-range amino acid interactions in ACC. And through the AC, which is based on the physicochemical properties, the local features of amino acids with adjacent effects were extracted, and more comprehensive protein information was obtained, which provided strong support for the downstream feature extraction.

#### Amino acid composition (AAC)

The amino acid composition method [34] normalizes the frequency of occurrence of each amino acid in the protein, which is a concise protein feature extraction method. Specifically, the frequency of twenty amino acids in protein sequences is counted, and each protein sequence is converted into a 1 × 20-dimensional feature vector. The feature extraction formula is as follows:

1 $$ \mathrm{P}(x)=\frac{n}{N}  $$

Where *n* represents the number of amino acid *x* in the protein sequence and *N* represents the number of all amino acids in the protein.

#### Conjoint triad (CT)

The combined triplet method [27] takes an amino acid and its left and right amino acids as a unit, and divides 20 amino acids into 7 different clusters [19] according to the volume of amino acid side chains and dipoles (as shown in Table 2). Among them, different amino acids belonging to a certain cluster are considered to be the same. Therefore, the obtained feature is a 343-dimensional feature vector, which is the normalized results of triples (7*7*7). The formula is:

2 $$ \mathrm{P}(C)=\frac{N_{C}}{N-2}  $$

**Table 2:** Classification of amino acids based on amino acid side chains and dipole volume

Cluster	Amino acid
1	A, G, V
2	I, L, F, P
3	Y, M, T, S
4	H, N, Q, W
5	R, K
6	D, E
7	C

Among them, *C* represents a triplet, *N*_*C*_ represents the number of occurrences of this triplet, *N* represents the number of all amino acids in the protein, and the denominator represents that a protein sequence has *N*−2 triplets.

#### Auto covariance (AC)

The autocovariance method [37] mainly considers the proximity effect of amino acids. The interaction between amino acids and a fixed number of surrounding amino acids showed hydrophobicity (H1), hydrophilicity (H2), net charge index (NCI), Polarity (P1), polarizability (P2), soluble surface area (SASA), and side chains. The amino acid sequence is replaced by the initial values of the seven physical and chemical properties, and normalized to zero mean and unit standard deviation (SD), as shown in Formula (3).

3 $$ F_{i j}=\frac{f_{i, j}-f_{j}}{S_{i}}  $$

Where *f*_*i*,*j*_ represents the value of the *j*-th property of the *i*-th amino acid, *f*_*j*_ represents the average value of the *j*-th property of 20 amino acids, and *S*_*i*_ represents the corresponding standard deviation. The formula for calculating AC is as follows.

4 $$ \begin{array}{c} A C_{\text {lag,j }}=\frac{1}{N-\text { lag }} \sum_{i=1}^{N-l a g}\left(F_{i j}-\frac{1}{N} \sum_{i=1}^{N} F_{i j}\right) \times\\ \left(F_{(i+l a g), j}-\frac{1}{N} \sum_{i=1}^{N} F_{i j}\right) \end{array}  $$

Among them, *lag* represents the distance between the residuals, and *N* represents the length of the protein sequence. In this paper, *j* takes 7 to represent 7 physical and chemical properties. When the *lag* is taken as 30, it can not only avoid the difficulty of capturing useful protein sequence features due to too close amino acid distance but also solve the problem of noise caused by too much amino acid content[20].

### PPIs model based on self-attention combined with deep neural network

**Fig. 1:**
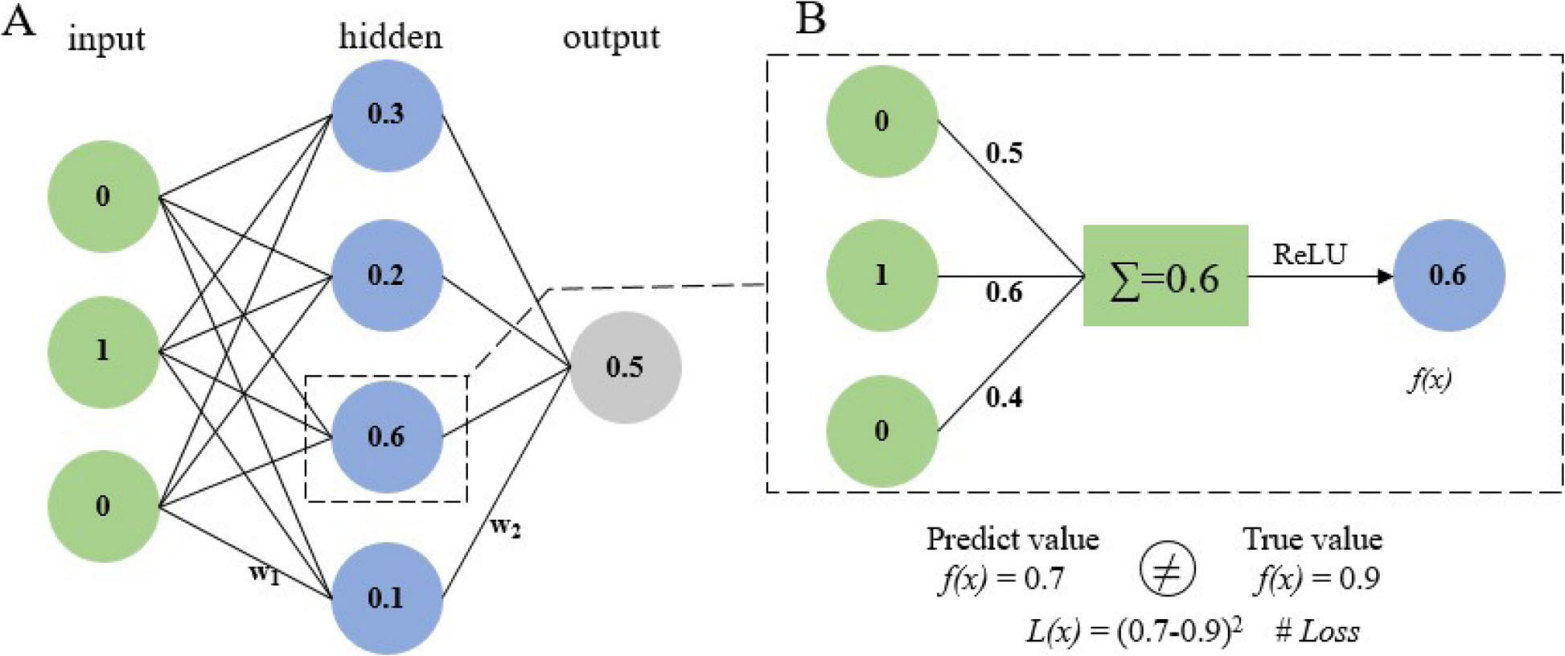
Neural network procedure

**Fig. 2:**
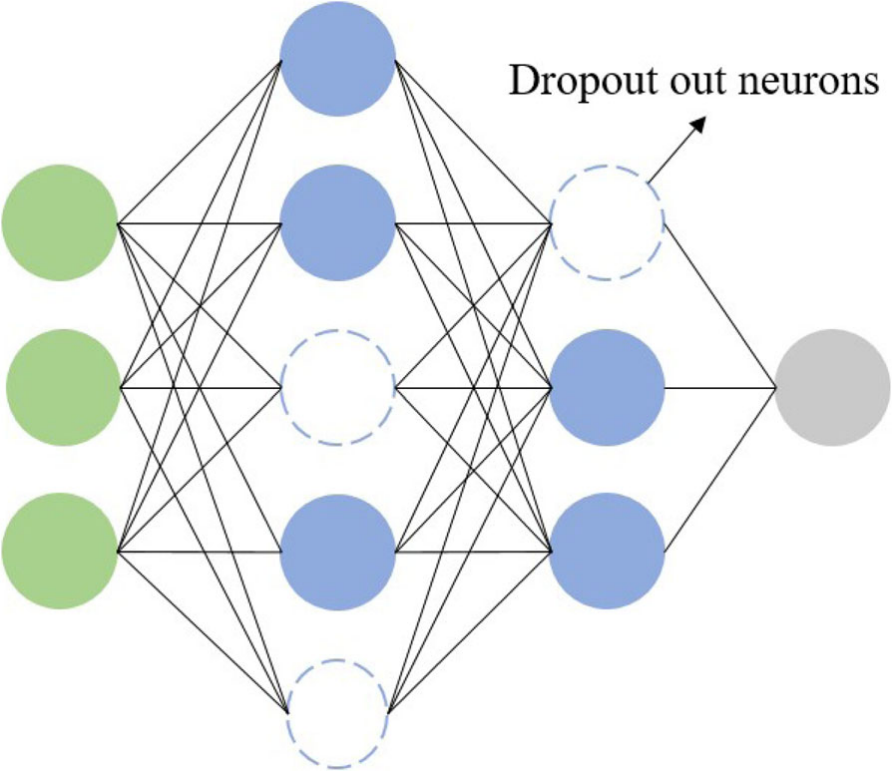
Networks with dropout

The simple neural network receives data at the input layer, transforms the data through multiple hidden layers, and finally computes the result at the output layer. Neurons in the hidden or output layer are connected to all neurons in the previous layers, as shown in Fig. 1A. Each neuron computes a weighted sum of its inputs and applies a nonlinear activation function to compute its output *f*(*x*) (Fig. 1B). The most commonly used activation function is the Rectified Linear Unit (ReLU), which sets the negative signal threshold to 0 and allows positive signals to pass normally. The deep neural network (DNN) proposed in recent years is an artificial neural network inspired by the neural network of the brain, which consists of multiple interconnected computing units (neurons) and extracts high-level abstractions from data. DNN is widely used in speech recognition, PPIs [34], and other fields with its powerful feature extraction ability. DNN takes the received data as input, then transforms it in a non-linear way, and the last layer outputs [43, 44]. With regard to avoid over fitting, a dropout layer is also added to drop some neurons during training, as shown in Fig. 2.

**Fig. 3:**
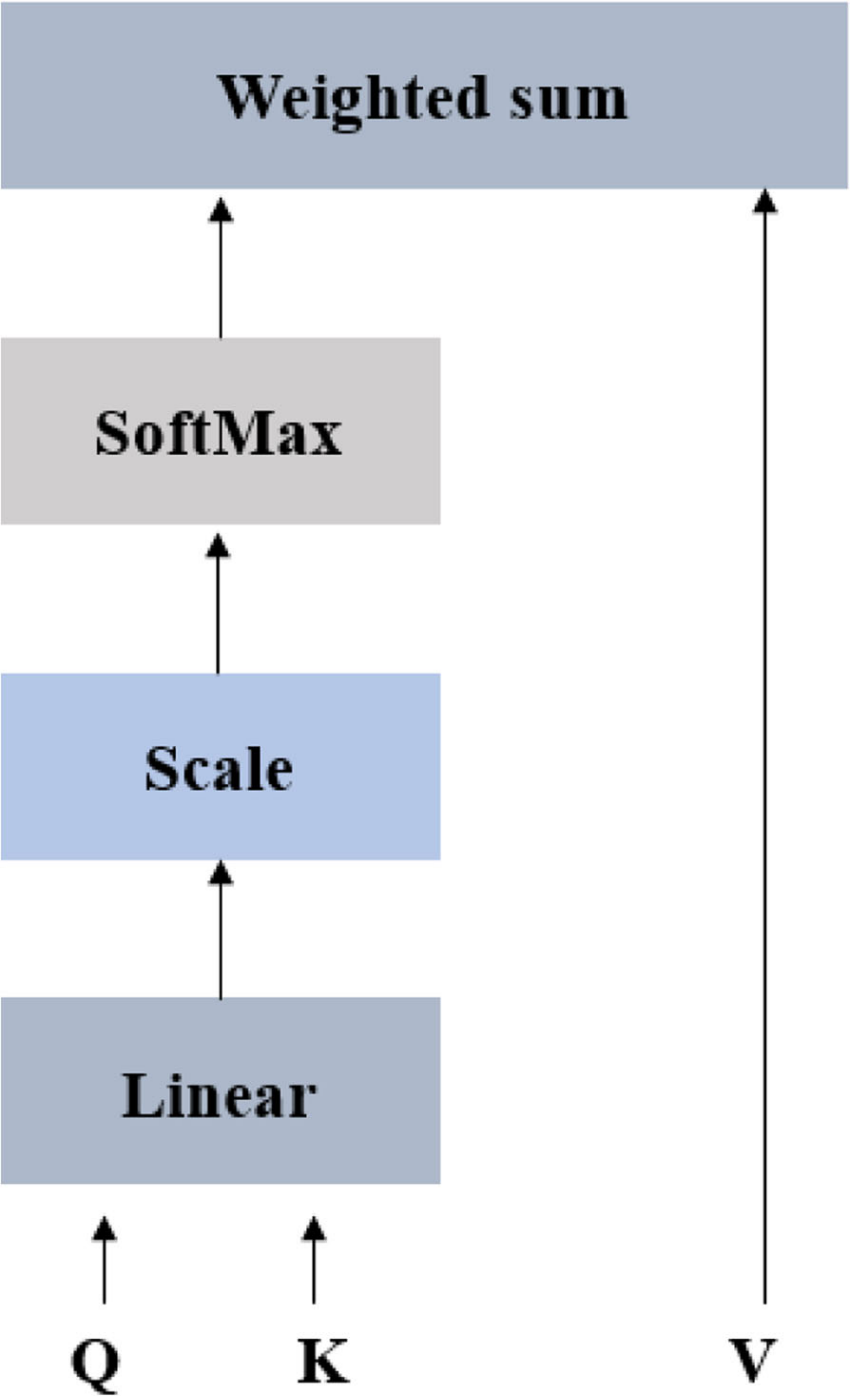
Model of Self-attention

Self-attention mechanism (Fig. 3) is a model framework proposed by the Google team [45] in 2017, which can reduce the dependence on external information and be better at capturing the internal correlation of data or features, especially long-distance dependency[46]. As shown in Fig. 3, the weight is obtained by calculating the similarity of *Q* and *K* after linear transformation, then the softmax function is used to normalize the weight, and finally attention is obtained by the weight and *V*.Then, the output of the self-attention module is the weighted sum of feature vectors on all the amino acids, and its core formula is :

5 $$ \operatorname{Attention}(Q, K, V)=\operatorname{softmax}\left(\frac{Q K^{T}}{\sqrt{d}_{k}}\right) V  $$

Where *d*_*k*_ square root represents the scaling factor to control the magnitude of the dot product. *Q*, *K* and *V* represent the query, key and value of the amino acid, respectively.

**Fig. 4:**
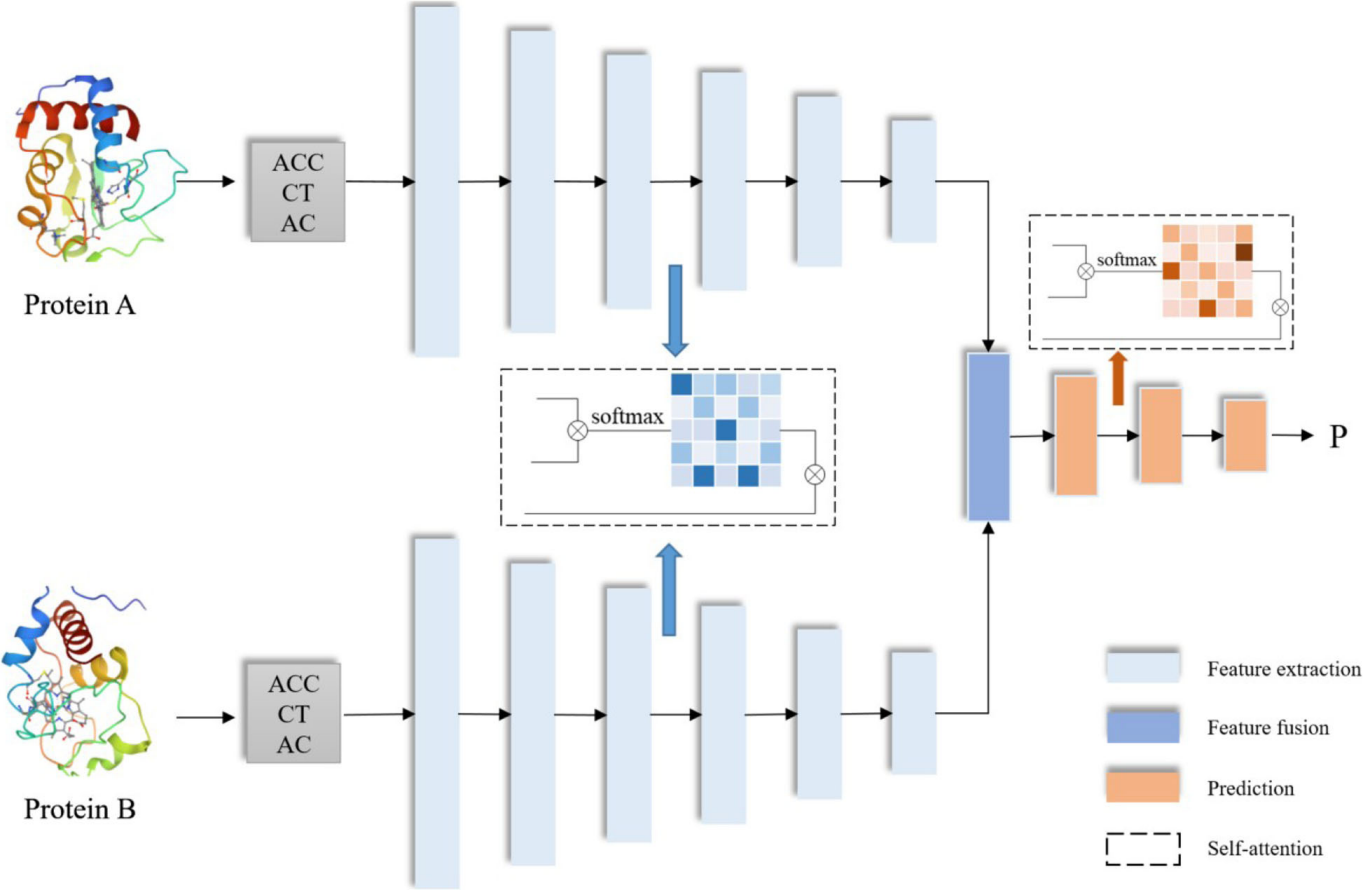
SDNN-PPI for prediction of protein-protein interaction

Based on the excellent performance of deep neural network and Attention mechanism, this paper proposes a DNN network that applies multi-layer fully connected layers and self-attention to predict PPIs, named SDNN-PPI. Deep networks have the characteristics of synthesizing various information, but as the number of layers increases, the risk of overfitting will increase, and the focus on key data will also be reduced. Therefore, this paper dynamically pays attention to the key residues in the sequence through the self-attention in the feature extraction layer, adjusts the weights, captures the feature of single residue, promotes the prediction process, and avoids falling into local optimum caused by DNN overfitting. In addition, since self-attention has a strong ability to extract internal features, it is widely used to capture long-range dependencies between tokens in sequential data. Therefore, in the prediction stage, self-attention mechanism is used to enhance the feature extraction of protein pairs, and further exploits the potential relationship of residues to obtain more accurate information. The SDNN-PPI model is shown in Fig. 4. It mainly includes three modules, namely the feature extraction layer, the feature fusion layer, and the PPIs prediction layer.

(1) Input layer: The model is based on two proteins (P1, P2) as input, and converts the protein sequences into feature vectors through the three encoding methods of AAC, CT, and AC. Finally, each protein sequence is encoded into a vector with dimension of 573, which consists of 20 AAC features, 343 CT features, and 210 AC features respectively.

(2) Feature extraction layer: SDNN-PPI is composed of two channels, which extract the hidden information of proteins respectively. Each channel is composed of six fully connected layers (1024-512-256-128-64-32) by adding a self-attention layer that adjusts the global weight of the sequence. To avoid gradient vanishing and over fitting, Batch Normalization and Dropout layers are added after each dense layer. The formula is expressed as:

6 $$ f=\operatorname{Dropout}(B N(\operatorname{Dense}(P)))  $$

Where *P* represents the feature vector of protein sequence, and *f* represents the output through the full connection layer.

(3) Feature fusion layer: The feature fusion layer connects the protein information (F1’, F2’) obtained by the two channels from the feature extraction layer. The formula is expressed as:

7 $$ F=\operatorname{cat}\left(f 1^{\prime}, f 2^{\prime}\right)  $$

(4) Prediction layer: The prediction layer is composed of three fully connected layers (32-16-8) and a self-attention layer. Self-attention layer is conducive to increasing the exploration of protein pairs, which is put after the first dense layer. Then there is a single neuron with a Sigmoid activation function that converts the input from the previous layer into an output score. The formula is as follows:

8 $$ \mathrm{P}(P 1, P 2)=s(\operatorname{Dens}(F))  $$

where *s* denotes dense layer with one unit activated by sigmoid function.

### Evaluation metrics

The following assessments are used for this article: Accuracy (ACC), Sensitivity (Sens), Specificity (Spec), Precision (Prec), Matthews Correlation Coefficient (MCC), and AUC. These assessments are used to calculate accuracy and bias to assess the feasibility and robustness of PPI forecasting methodologies. The definition formula is as follows:

9 $$ {}\text{Accuracy }=\frac{T P+T N}{T P+T N+F P+F N}  $$

10 $$ {}\text{Sensitivity }=\frac{T P}{T P+F N}  $$

11 $$ {}\text{Specificity }=\frac{T N}{T N+F P}  $$

12 $$ {}\text{Precision }=\frac{T P}{T P+F P}  $$

13 $$ {}\text{MCC}=\frac{T P \times T N-F P \times F N}{\sqrt{(T P+F P)(T P+F N)(T N+F P)(T N+F N)}}  $$

Among them, TP (True Positive) is the number of correctly predicted protein pair interactions in the sample data set, TN (True Negative) is the number of correctly predicted protein pairs that do not interact, FP (False Positive) is the number of non-interacting protein pairs predicted as interacting, while FN (False Negative) is the number of interacting protein pairs predicted as non-interacting.

In order to prove the statistical significance of SDNN-PPI, kappa coefficient[47] is also added. Kappa coefficient is an indicator to measure the consistency of two variables[48], which can be used to evaluate the classification accuracy. The results of kappa coefficient are usually between 0 and 1[49]. When the result is in the range of 0.0 to 0.20, the classification result is considered to be slight, kappa=0.21-0.40 means fair, kappa=0.41-0.60 is moderate, kappa=0.61-0.80 describes substantial, and kappa > 0.81 represents almost perfect. Its calculation formula[47] is as follows:

14 $$ kappa=\frac{p_{0}-p_{e}}{1-p_{e}}  $$

Where *p*_0_ means accuracy,

15 $$ {}p_{e}=\frac{(T N+F P)(T N+F N)}{(T P+F P+T N+F N)^{2}}+\frac{(F N+T P)(F P+T P)}{(T P+F P+T N+F N)^{2}}  $$

## Results and discussion

This part mainly evaluates and discusses the performance of the model. Firstly, the coding method used in this work is described, which can achieve ideal results. Secondly, the framework of the model is determined. Then, the results for two intraspecific and interspecific datasets. Subsequently, SDNN-PPI and existing advanced algorithms are compared on intraspecies and interspecific datasets to evaluate the validity of the model. Then, four independent data sets are used to prove the robustness of the model. Finally, the PPI networks further prove the potential capability of the model in predicting disease development.

### Encoding method selection

In this paper, encoding methods of ACC, CT and AC were used to construct 573-dimensional feature vectors to encode proteins, which can extract global and local features. In addition, LD was also used to encode local characteristics of proteins [27]. LD can encode each protein sequence into a 630-dimensional vector. In order to verify the encoding scheme, LD was also originally used in our experiments as another optional encoding method for protein pairs, and S.cerevisiae (core subset) data set was selected to search for best encoding combination scheme based on the experimental results of the model. In order to exclude the influence of the superiority of the SDNN-PPI model on the results, the standard two-channel self-attention model was selected to verify the encoding scheme. The two-channel self-attention model, which is used for encoding methods selection, is very concise. The input proteins A and B were encoded into feature vectors by the method in the first column of Table 3. Then the two vectors were input to two identical feature extraction layers, which only adopted the self-attention mechanism. Then, feature fusion is performed on the two protein vectors extracted from feature extraction, and the final result is obtained under the action of fully connected prediction layers. As shown in Table 3, compared with the other 10 combination schemes, the ACC+CT+AC encoding combination scheme achieved the optimal results on 6 evaluation indicators. However, after the addition of LD in encoding scheme ACC+CT+AC, the results did not improve effectively, which may be due to the fact that LD was not accurate enough to extract the features of the encoding of excessively long protein sequences, resulting in poor effects.

**Table 3:** Performance of different coding methods

Encoding methods	Length	ACC(%)	Spec(%)	Sens(%)	Prec(%)	MCC(%)	AUC(%)
ACC+CT+LD+AC	1203	92.31 ± 0.66	94.37 ± 0.25	90.26 ± 1.28	94.13 ± 0.25	84.70 ± 1.27	97.00
ACC+CT+LD	993	92.00 ± 0.66	93.31 ± 1.04	90.69 ± 0.57	93.14 ± 1.01	84.03 ± 1.33	97.03
ACC+CT+AC	573	**95.19 ± 0.68**	**97.05 ± 0.65**	**93.33 ± 1.20**	**96.94 ± 0.65**	**90.45 ± 1.34**	**98.60**
ACC+LD+AC	860	91.41 ± 0.52	93.06 ± 0.87	89.76 ± 0.64	92.83 ± 0.86	82.87 ± 1.06	96.58
CT+LD+AC	1183	89.50 ± 0.68	90.97 ± 0.58	88.02 ± 1.47	90.70 ± 0.50	79.04 ± 1.33	95.62
ACC+CT	363	89.79 ± 0.65	90.79 ± 0.81	88.79 ± 0.87	90.61 ± 0.76	79.61 ± 1.29	95.78
ACC+LD	650	88.93 ± 0.43	89.67 ± 1.37	88.20 ± 1.53	89.54 ± 1.14	77.91 ± 0.88	95.05
ACC+AC	230	85.74 ± 1.80	87.20 ± 1.74	84.29 ± 2.66	86.82 ± 1.74	71.54 ± 3.60	92.59
CT+LD	973	90.47 ± 0.52	92.76 ± 0.57	88.18 ± 0.93	92.42 ± 0.55	81.03 ± 1.02	95.97
CT+AC	553	89.06 ± 0.55	90.70 ± 0.87	87.43 ± 1.34	90.40 ± 0.73	78.19 ± 1.07	94.74
LD+AC	840	91.44 ± 0.44	92.72 ± 0.72	90.17 ± 0.56	92.54 ± 0.69	82.92 ± 0.89	96.60

### Model ablation experiment

To verify the effect of different network structures on the performance of SDNN-PPI, two different network structures were first designed. (a) using a dual-channel network to extract protein information (DNN-PPI a),which is the SDNN-PPI model without the self-attention part. And (b) directly connect two proteins in a single channel network (DNN-PPI b). As can be seen from the first two lines of Table 4, the dual-channel model was superior to the single-channel model, and the ACC, Spec, Sens, Prec, MCC, and AUC values of DNN-PPI a were 3.12%, 2.79%, 5.84%, 2.92%, 6.22%, and 1.49% higher than those of DNN-PPI b, respectively. Secondly, after setting up the dual-channel model, the meaning of Self-attention was studied. The following was the control variable method based on SDNN-PPI. (c) self-attention was added in feature extraction layer (SDNN-PPI a), (d) self-attention was added in prediction layer (SDNN-PPI b), (e) self-attention was added in both feature extraction layer and prediction layer (SDNN-PPI), (f) dual-channel network without self-attention (DNN-PPI a). After building different networks, the S.cerevisiae (core subset) dataset was used to evaluate the model results. As shown in Table 4, the SDNN-PPI performed better, so this model was chosen as the final framework.

**Table 4:** Comparison among different layer architectures for SDNN-PPI on S.cerevisiae(core subset)

Architectures	ACC(%)	Spec(%)	Sens(%)	Prec(%)	MCC(%)	AUC(%)
DNN-PPI a	94.9	96.35	95.83	96.25	89.84	98.54
DNN-PPI b	91.78	93.56	89.99	93.33	83.62	97.05
SDNN-PPI a	95.16	96.96	93.37	96.86	90.4	98.53
SDNN-PPI b	95.21	96.98	93.44	96.87	90.48	98.56
SDNN-PPI	**95.48**	**97.23**	**93.80**	**97.13**	**91.02**	**98.63**

### Performance of the SDNN-PPI

When training a model with dataset, it is easy to overfit due to unreasonable division of the dataset. Compared with the division technique of traditional models (dividing fixed training sets and test sets), cross-validation can avoid such problems, so this paper uses the 5-fold cross-validation method to evaluate the model. The experimental data is randomly divided into 5 parts, samples of 4 parts are randomly taken as the training set, the other part is used as the test set, and finally the average of the 5 test sets is calculated. Table 5, 6, 7 and 8 presented the cross-validation results of this method. In addition, the performance of this method was compared with several advanced methods, and the results were shown in Table 10, 11, 12 and 13.

**Table 5:** Prediction results of S.cerevisiae (core subset) under five-fold cross-validation

testing set	ACC(%)	Spec(%)	Sens(%)	Prec(%)	MCC(%)	AUC(%)
1	95.44	97.59	93.48	97.48	90.97	98.42
2	95.17	96.69	94.1	96.59	90.39	98.92
3	95.13	97.32	93.3	97.20	90.35	98.28
4	95.49	96.37	94.36	96.27	91.98	98.74
5	96.16	98.21	93.74	98.14	92.39	98.80
average	95.48 ± 0.37	97.23 ± 0.66	93.80 ± 0.39	97.13 ± 0.66	91.02 ± 0.74	98.63

**Table 6:** Prediction results of Human data set under five-fold cross-validation

Testing set	ACC(%)	Spec(%)	Sens(%)	Prec(%)	MCC(%)	AUC(%)
1	98.78	99.49	98.08	98.97	97.06	99.67
2	99.29	98.85	99.49	99.23	98.46	99.63
3	98.85	99.10	98.46	98.97	97.56	99.51
4	98.78	99.36	98.72	99.1	97.95	99.72
5	98.97	98.84	99.10	98.83	96.8	99.46
average	98.94 ± 0.19	99.10 ± 0.24	98.77 ± 0.49	99.02 ± 0.13	97.57 ± 0.60	99.60

**Table 7:** Prediction results of Human-B.Anthracis data set under five-fold cross-validation

Testing set	ACC(%)	Spec(%)	Sens(%)	Prec(%)	MCC(%)	AUC(%)
1	91.44	85.14	97.74	86.8	83.54	97.93
2	93.78	93.05	94.51	93.15	87.57	98.65
3	92.49	87.72	97.25	88.79	85.36	98.03
4	94.26	90.78	97.74	91.39	88.73	98.22
5	93.78	91.76	95.79	92.07	87.62	98.32
average	93.15 ± 1.03	89.69 ± 2.88	96.61 ± 1.27	90.44 ± 2.32	86.57 ± 1.87	98.23

**Table 8:** Prediction results of Human-Y.pestis data set under five-fold cross-validation

Testing set	ACC(%)	Spec(%)	Sens(%)	Prec(%)	MCC(%)	AUC(%)
1	85.91	75.98	95.85	79.94	73.28	95.73
2	88.16	83.9	92.43	85.15	76.61	95.39
3	88.16	80.83	95.49	83.3	77.16	95.73
4	91.27	89.26	93.29	89.68	82.62	96.31
5	88.16	83.76	92.55	85.07	76.61	95.55
average	88.33 ± 1.71	82.74 ± 4.34	93.92 ± 6.06	84.63 ± 1.85	77.26 ± 3.79	95.74

As can be seen from Table 5, SDNN-PPI had an excellent prediction performance for intraspecific data sets. The average prediction results of S.cerevisiae (core subset) in ACC, Spec, Sens, Prec, MCC and AUC were 95.48%, 97.23%, 93.80%, 97.13%, 91.02% and 98.63%, respectively. Similarly, the average results of the Human dataset were ACC 98.94%, Spec 99.10%, Sens 98.77%, Prec 99.02%, MCC 97.57%, and AUC 99.60%, as shown in Table 6. Meanwhile, for the interspecific data set, as shown in Table 7–8, SDNN-PPI achieved 93.15% and 88.33% accuracy in Human-B.anthracis and Human-Y.pestis, respectively. The above experimental results show that the prediction of PPIs by SDNN-PPI is effective and robust. Table 9 presented the statistical significance of SDNN-PPI in four data sets. According to the above description, kappa between 0.61-0.80 indicates that the classification results were substantial, and when kappa > 0.81, the classification results were almost perfect. The kappa values of the 4 data sets in Table 9 were all greater than 0.61 and 3 were greater than 0.81, indicating that the results were statistically significant.

**Table 9:** Prediction results of four data sets in kappa coefficient

Data sets	S.cerevisiae(core subset)	Human	Human-B.Anthracis	Human-Y.pestis
kappa	0.91	0.98	0.85	0.76

### Compared with other methods

To predict protein-protein interactions, various prediction methods have been continuously proposed. In order to more objectively evaluate the predictive performance of the constructed model, the prediction results were compared with other models in the same data set. The comparison results of the intraspecific datasets S.cerevisiae (core subset) and Human were shown in Tables 10 and 11. The interspecific datasets Human-B.Anthracis and Human-Y.pestis results were shown in Tables 12 and 13. For comparison methods, the data in the table were extracted from the original text, and N/A means that the data is not available in the original text. And the values in bold indicate the optimal value for this column.

**Table 10:** Comparison results of different PPIs prediction methods on S.cerevisiae (core subset)

Methods	ACC(%)	Spec(%)	Sens(%)	Prec(%)	MCC(%)	AUC(%)
DeepPPI[34]	94.43 ± 0.30	N/A	92.06 ± 0.36	96.65 ± 0.59	88.97 ± 0.62	N/A
DeepFE-PPI[39]	94.78 ± 0.61	N/A	92.99 ± 0.66	96.45 ± 0.87	89.62 ± 1.23	N/A
LightGBM-PPI[27]	95.07	97.94	92.21	97.82	90.30	N/A
Bio2Vec[59]	93.30	N/A	92.70	93.55	87.49	97.20
StackPPI[50]	94.64	96.46	92.81	96.33	89.34	N/A
GTB-PPI[28]	95.15 ± 0.25	N/A	92.21 ± 0.36	97.97 ± 0.60	90.45 ± 0.53	N/A
AE-LGBM[60]	95.40 ± 0.20	**98.70 ± 0.20**	92.10 ± 0.30	N/A	91.00 ± 0.40	N/A
GcForest-PPI[41]	95.44 ± 0.18	N/A	92.72 ± 0.44	**98.05 ± 0.25**	91.02 ± 0.35	N/A
SDNN-PPI	**95.48 ± 0.37**	97.23 ± 0.66	**93.80 ± 0.39**	97.13 ± 0.66	**91.02 ± 0.74**	**98.63**

**Table 11:** Comparison results of different PPIs prediction methods on Human

Methods	ACC(%)	Spec(%)	Sens(%)	Prec(%)	MCC(%)	AUC(%)
RPEC[26]	96.59	N/A	96.72	96.18	93.18	N/A
Bio2Vec[59]	97.31	N/A	96.28	98.48	94.76	**99.61**
GWOSVM[61]	94.56	N/A	95.55	93.08	89.51	N/A
DeepFE-PPI[34]	98.71 ± 0.30	N/A	98.54 ± 0.55	98.77 ± 0.53	97.43 ± 0.61	N/A
AE-LGBM[60]	98.70 ± 0.10	**99.20 ± 0.20**	98.10 ± 0.20	N/A	97.30 ± 0.30	N/A
AE-AC[60]	97.19	98.06	96.34	N/A	N/A	N/A
SDNN-PPI	**98.94 ± 0.19**	99.10 ± 0.24	**98.77 ± 0.49**	**99.02 ± 0.13**	**97.57 ± 0.60**	99.60

**Table 12:** Comparison results of different PPIs prediction methods on Human-B.Anthracis

Methods	ACC(%)	Sens(%)	Prec(%)	MCC(%)	AUC(%)
LBE-BN[61]	78.70	73.00	42.00	43.40	83.70
LBE-NB[61]	82.50	53.80	47.80	39.70	82.10
LBE-RF[61]	85.40	24.0	67.00	34.00	86.80
ACC-BN[61]	77.40	51.70	37.30	30.30	79.00
LBE-j48[61]	80.06	31.20	39.60	23.90	54.10
LD-DNN[42]	91.70	89.50	**93.90**	83.50	96.37
SDNN-PPI	**93.15**	**96.61**	90.44	**86.57**	**98.23**

**Table 13:** Comparison results of different PPIs prediction methods on Human-Y.pestis

Methods	ACC(%)	Sens(%)	Prec(%)	MCC(%)	AUC(%)
LBE-BN[61]	76.10	73.50	38.60	40.10	81.30
LBE-NB[61]	80.90	45.50	43.2	32.80	78.60
LBE-RF[61]	84.6	16.00	66.30	27.30	83.50
ACC-BN[61]	80.00	52.40	42.10	34.90	75.60
LBE-j48[61]	80.10	27.90	37.10	20.80	51.70
LD-DNN[42]	87.30	84.20	**90.40**	74.90	94.99
SDNN-PPI	**88.33**	**93.92**	84.63	**77.26**	**95.74**

As can be seen from Table 10, ACC, Spec, Sens, Prec, MCC, and AUC of SDNN-PPI were 95.48%, 97.23%, 93.80%, 97.13%, 91.02% and 98.63%, respectively. Compared with other methods, its ACC increased by 0.04% 2.18%. According to Table 11, ACC, Spec, Sens, Prec, MCC, and AUC of SDNN-PPI in Human data set were 98.94%, 99.10%, 98.77%, 99.02%, 97.57% and 99.60%, respectively. Compared with other methods, the accuracy of this method is obviously improved. Although SDNN-PPIs was not optimal in all indicators, it was higher in more than half of the indicators on S.cerevisiae (core subset) and human datasets, indicating that the method was still competitive. For this, the predictive performance of SDNN-PPI method became significantly better than other methods in multiple indicators.

As can be seen from Table 12, ACC, Sens, Prec, MCC and AUC of SDNN-PPI in Human-B.anthracis data set are 93.15%, 96.61%, 90.44%, 86.57% and 98.23%, respectively. The ACC of SDNN-PPI is 93.15%, which is significantly higher than other methods. According to Table 13, the ACC, Sens, Prec, MCC and AUC of SDNN-PPI in Human-Y.pestis data set were 88.33%, 93.92%, 84.63%, 77.26% and 95.74%, respectively. In comparison to other methods, its ACC value was 1.03% ∼ 12.23% higher than other methods. Therefore, the SDNN-PPI method achieves comparative results on interspecies datasets. It was worth noting that the two tables do not display Spec columns because the models being compared did not have Spec values.

### Performance on independent data sets

In order to further verify the generalization ability of SDNN-PPI, Saccharomyces cerevisiae [34] was selected as the training set, and C.elegans, E.coli, H.sapiens and M.musculus were selected as independent test sets. The number of interaction pairs of the independent test set was shown in the test pairs in the Table 14. In addition, the results were evaluated by ACC. Saccharomyces cerevisiae set consists of 17257 positive pairs and 48594 negative pairs, from which the same number of positive and negative samples are randomly selected to train the model. The prediction results were shown in Table 14. As can be seen from Table 14, the accuracy of SDNN-PPI in these four independent data sets was 100%. This can show that SDNN-PPI achieved good predictive performance on four independent test sets, indicating that the proposed model can characterize important PPIs information and make cross-species predictions. In other words, PPIs prediction models generated by one species can be migrated to other species.

**Table 14:** Comparison of ACC of different PPIs prediction methods on independent test sets

Methods/Species	C.elegans	E.coli	H.sapiens	M.musculus
test pairs	4013	6984	1412	313
DeepPPI[34]	94.84	92.19	93.77	91.37
DeepFE-PPI[39]	**100**	**100**	**100**	**100**
LightGBM-PPI[27]	90.16	92.16	94.83	94.57
StackPPI[50]	97.11	98.71	97.66	98.40
GcForest-PPI[41]	96.01	96.3	98.58	99.04
GTB-PPI[28]	92.42	94.06	97.38	98.08
AE-LGBM[60]	90.10	92.10	94.80	94.50
SDNN-PPI	**100**	**100**	**100**	**100**

### Performance on PPI networks

Studying the network of PPIs [28] is also of great significance to understanding other information about proteins, and the corresponding biological topological properties can be studied. In this paper, SDNN-PPI detected two important PPIs networks, namely the one-core network and crossover network of Wnt-related pathway. The mononuclear PPIs network is a network of PPIs composed of a core protein, CD9 [41], and interacts with many other proteins. CD9 is a tetrameric protein that plays an important role in cell viability and tumor suppression. The network is composed of CD9 as the core protein and 17 other genes.

**Fig. 5:**
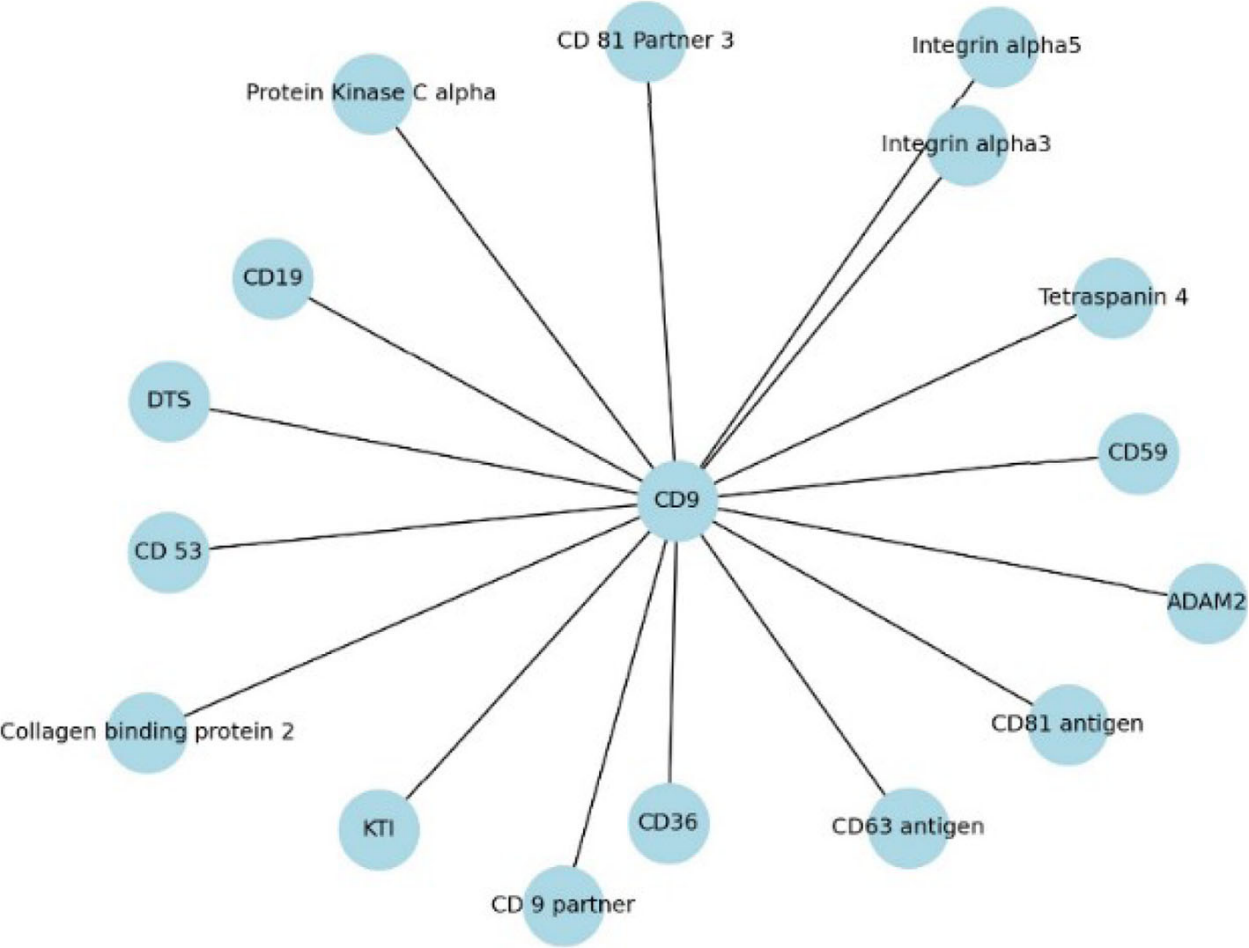
The predicted results of PPIs networks of a one-core network for CD9

**Fig. 6:**
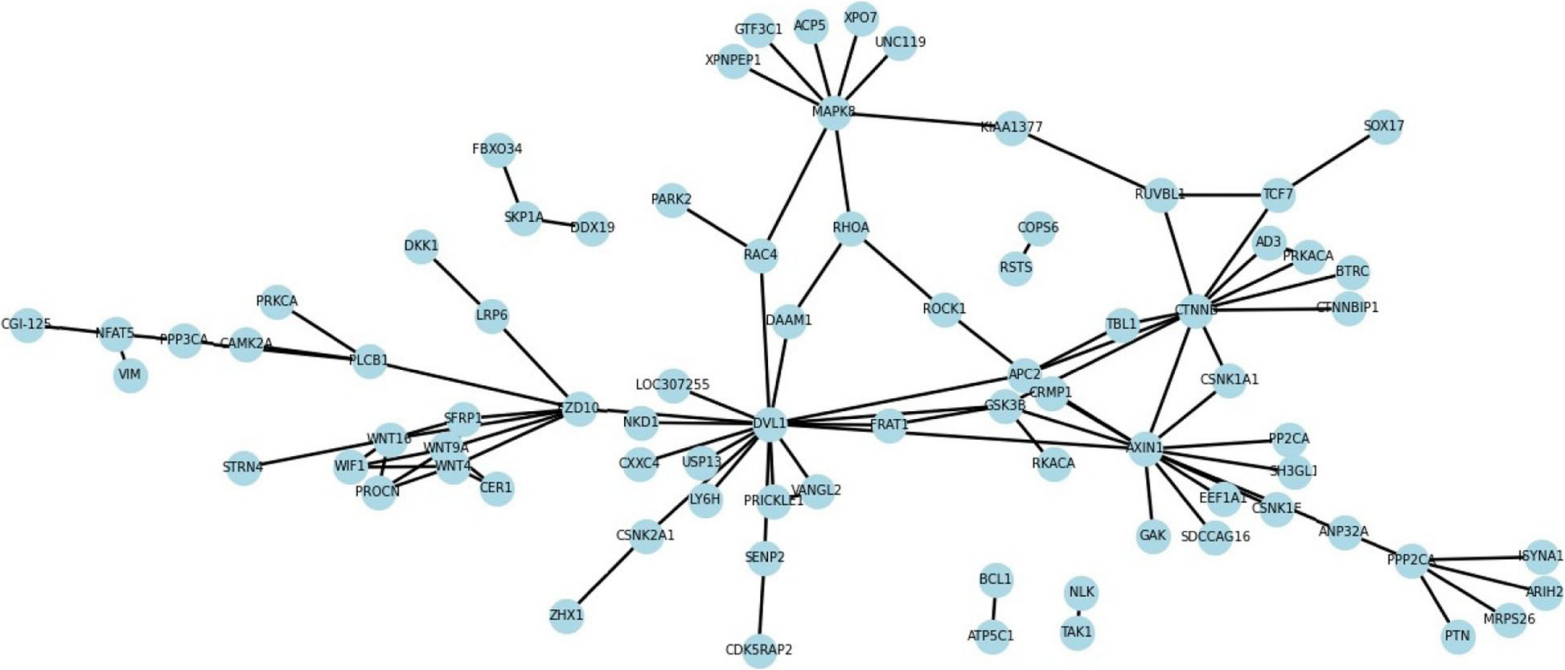
The predicted results of a crossover network for the Wnt-related pathway

The second is a typical crossover and multicore network [50] constructed by 78 genes. This pathway network plays a crucial role in tumor growth and tumor formation. AAC, CT, and AC were used to encode proteins to obtain a 573-dimensional feature vector. The Saccharomyces cerevisiae dataset was used as the training set, and the one-core network and crossover network of the wnt-related paths were used as the test set. The one-core network prediction results of the wnt-related paths were shown in Fig. 5, and the other in Fig. 6. Solid lines represent true predictions and dashed lines represent false predictions. It can be obtained from the graph that all interacting proteins are correctly identified. Table 15 showed the prediction results of various methods on the two network datasets. The results shown that the proposed method produces comparable or better results in comparison to existing models. After the above discussion, SDNN-PPI was a model with high generalization ability, which can obtain competitive results in multiple data sets and effectively improve the prediction accuracy of PPIs.

**Table 15:** Performance of different methods on PPI network

	LightGBM-PPI[27]	StackPPI[50]	GTB-PPI[28]	AE-LGBM[60]	GcForest-PPI[41]	SDNN-PPI
CD9	15/16	N/A	15/16	16/16	16/16	**16/16**
Wnt	89/96	93/96	92/96	95/96	94/96	**96/96**

## Conclusion

The study of PPIs is of great significance for understanding cellular regulation and signal transduction, as well as for exploring and elucidating the mechanism of protein interactions in cells. In this paper, we proposed SDNN-PPI, a self-attention-based deep learning neural network prediction method for PPIs. The protein sequences were encoded by AAC, CT and AC methods, and excellent accuracy was obtained in the intraspecific data sets (S.cerevisiae core subset and Human) and interspecies data sets (Human-B.anthracis and Human-Y.pestis). In order to further verify the universality of SDNN-PPI, the evaluation of C.elegans, E.coli, H.sapiens and M.musculus data sets also achieved competitive accuracy, indicating that the method can also achieve good performance in cross-species prediction. The PPI network prediction based on one-core and crossover network correctly predicted the protein interaction containing cell and tumor information on the network. Therefore, comprehensive evaluations demonstrated that SDNN-PPI method could provide a new way to solve problems in signaling pathway research, drug-target prediction and disease pathogenesis research [51–54]. Although protein sequences are transformed into vectors through various encoding methods, the acquisition of comprehensive protein characteristic information is still insufficient. How to better mine the structural information, evolutionary information set of protein pairs and the relationship between protein residues is leading us to the next research direction. At the same time, DNA computing and DNA storage [55, 56] have been applied in more fields [57, 58], and the storage of known protein information and structure may also play a role in promoting biological evolution.

## Data Availability

The data and code underlying this article are available in https://github.com/xueleecs/SDNN-PPI.The article all data set on the https://github.com/xueleecs/SDNN-PPI/tree/main/Data.

## Abbreviations

PPI: Protein-protein interactions
AAC: amino acid composition
CT: conjoint triad
AC: auto covariance
LD: local descriptor
MAC: Moran autocorrelation
PSSM: position specific scoring matrix
AD: autocorrelation descriptor
GTB-gradient tree boosting; DC: gradient tree boosting
LCTD: local composition ternary description
MCD: multi-scale continuous and discontinuous local descriptors
MMI: multivariate mutual information
CTD: composition-transition-distribution
DPC-PSSM: dipeptide composition PSSM
TP: true positives
TN: true negatives
FP: false positives
FN: false negatives
NB: Naive Bayes
DNN: Deep neural network
